# Use of oXiris^®^ vs standard AN69ST filters in sepsis-associated acute kidney injury requiring continuous renal replacement therapy: a retrospective matched cohort study

**DOI:** 10.3389/fneph.2026.1810007

**Published:** 2026-05-12

**Authors:** David Yepes Gómez, Sara Moreno-Bedoya, Sofía Lohle-Rueda, Juan Rodrigo Moreno-Restrepo, María Palacio-Muñoz, Nicolás Gómez-Correa, Andrea Sierra Ramirez, María Camila Salazar Agudelo, Esteban Villegas-Arbeláez

**Affiliations:** 1 Critical Care Physician, Department Head, Victoriana Clinic, Intensivist, Clínica Las Américas, Medellín, Colombia; 2 Postgraduate in Epidemiology, Anesthesiology resident, Faculty of Medicine, Universidad CES, Medellín, Colombia; 3 Master of Business Administration (MBA) candidate, Faculty of Medicine, Universidad del Rosario, Bogotá, Colombia; 4 Critical Care specialist, Critical Care Unit coordinator, Clínica CES, Medellín, Colombia; 5 General Practitioner, High Dependency Unit, Clínica CES, Medellín, Colombia; 6 Anesthesiology resident, Faculty of Medicine, Universidad CES, Medellín, Colombia; 7 Otorhinolaryngology resident, Faculty of Medicine, Universidad de Cartagena, Cartagena, Colombia; 8 Clinical Epidemiology, Health Research Lead, Clínica CES, Medellín, Colombia

**Keywords:** acute kidney injury, continuous renal replacement therapy, cytokine adsorption, hemofilter, oXiris, sepsis, septic shock, ventilator-free days

## Abstract

**Background:**

Sepsis-associated acute kidney injury (SA-AKI) is common in critically ill patients and carries high short-term mortality despite timely antimicrobials and organ support. Adsorptive hemofilters such as the oXiris^®^ membrane have been proposed as adjuncts during continuous renal replacement therapy (CRRT), but their patient-centered benefit remains uncertain.

**Methods:**

We performed a retrospective matched cohort study within a dynamic cohort of consecutive adults with septic shock and SA-AKI requiring CRRT in a tertiary ICU in Medellín, Colombia (November 2020 - May 2023). Exposure was the hemofilter used at CRRT initiation (oXiris^®^ vs standard AN69ST). Patients were matched 2:1 without replacement using nearest-neighbor propensity scores based on age, Sequential Organ Failure Assessment (SOFA) score at ICU admission, time from ICU admission to CRRT initiation, and infection source. The primary outcome was 28-day all-cause mortality from ICU admission. Secondary outcomes included ventilator-free days and ICU–free days at day 28, CRRT duration, and ICU length of stay. Prespecified sensitivity analyses excluded COVID-19 cases and used inverse probability of treatment weighting (IPTW). Exploratory analyses assessed early changes in SOFA, lactate, PaO2/FiO2, mean arterial pressure, vasopressor dose, and C-reactive protein.

**Results:**

After matching, 81 patients were included (53 standard filters and 28 oXiris^®^); 3 oXiris^®^- patients were unmatched. Twenty-eight-day mortality was 69.8% (37/53) with standard filters and 67.9% (19/28) with oXiris^®^. The adjusted risk ratio (RR) for oXiris^®^ was 0.95 (95% CI, 0.71-1.28; P = 0.76), with an absolute risk difference of -2.0% (95% CI, -23.2% to 19.3%). After excluding COVID-19 cases (n = 61), the adjusted RR was 1.05 (95% CI, 0.71-1.54; P = 0.81). IPTW analyses were directionally similar but considered secondary. Early physiologic changes showed no clear between-group differences.

**Conclusions:**

In adults with septic shock and SA-AKI requiring CRRT, oXiris^®^ use was not associated with lower 28-day mortality compared with standard AN69ST filters. Secondary and exploratory finding should be interpreted cautiously given residual confounding and data limitations. Randomized trials with standardized protocols are needed to identify potential benefiting subgroups.

## 1 Introduction

Sepsis and septic shock remain leading causes of intensive care unit mortality worldwide, and acute kidney injury is a frequent complication that increases the risk of prolonged organ support and death (1–4). In patients with septic shock and sepsis-associated acute kidney injury, continuous renal replacement therapy is often required for metabolic control and fluid management, yet short-term mortality remains high despite timely antimicrobials and protocolized supportive care (3, 5).

Persistent circulating inflammatory mediators and endotoxin have been proposed as contributors to ongoing organ dysfunction after infection control, motivating interest in extracorporeal blood purification strategies integrated into continuous renal replacement therapy (6–10). The oXiris^®^ membrane is a surface-treated AN69-based hemofilter designed to provide renal support while offering cytokine adsorption and enhanced endotoxin removal in a single circuit (10, 11).

Existing evidence suggests that adsorptive hemofiltration can influence physiological variables and inflammatory markers, but effects on patient-centered outcomes remain uncertain, and the overall certainty of evidence has been limited by small studies and risk of bias (9, 12). Most published oXiris^®^ data originate from high-income settings, while evidence from middle-income countries, where illness severity at presentation and resource constraints differ, remains sparse (5, 8).

We therefore evaluated whether oXiris^®^ use, compared with standard AN69ST filters, was associated with lower 28-day mortality among adults with septic shock and sepsis-associated acute kidney injury requiring continuous renal replacement therapy. Secondary aims were to describe competing-risk–aware duration outcomes and exploratory early physiologic changes available in the retrospective dataset.

## 2 Methods

### 2.1 Study design

This was a retrospective matched cohort study nested within a dynamic cohort of consecutive critically ill adults with septic shock and sepsis-associated acute kidney injury who required continuous renal replacement therapy in a tertiary academic intensive care unit.

### 2.2 Study setting and timeline

The study took place at CES Clinic, a tertiary academic medical center in Medellín, Colombia, serving as a referral hub for critically ill patients across the region. The institution has 210 adult hospital beds, including a 22-bed intensive care unit (ICU) with standardized protocols for the management of sepsis and renal replacement therapy. Patient recruitment occurred consecutively between November 2020 and May 2023, encompassing diverse etiologies of septic shock during and following the COVID-19 pandemic. Follow-up extended from ICU admission through day 28, regardless of ICU or hospital discharge status, to ascertain vital status. Data analysis was performed between July 2023 and March 2024.

### 2.3 Participants

Adults 18 years or older were eligible if they met 3 criteria: septic shock defined according to Sepsis-3 criteria (3), including persistent hypotension requiring vasopressors and serum lactate greater than 2 mmol/L despite adequate fluid resuscitation; acute kidney injury staged as KDIGO class II or III (13) and judged clinically to be sepsis-associated; and a clinical indication for CRRT determined by the treating intensivist. Exclusion criteria were chronic kidney disease with an estimated glomerular filtration rate below 60 mL/min/1.73 m² for more than 3 months before admission, advance directives limiting aggressive treatment, a terminal condition with expected survival below 48 hours, or inability to obtain consent for routine clinical use of the device and data collection according to local practice.

We identified all consecutive eligible patients during the study period. Exposure was defined as the hemofilter used at CRRT initiation and recorded as standard AN69ST or oXiris^®^. In our database, each patient contributed a single exposure category at treatment initiation. The analytic dataset did not systematically capture crossover between membranes, number of sequential filters, or filter-life variables; accordingly, treatment was analyzed according to filter assignment at CRRT initiation. oXiris^®^ selection reflected clinician judgment and device availability in patients perceived to have greater inflammatory burden or hemodynamic instability, whereas standard AN69ST filters were used when adsorptive therapy was not selected or not available.

Cases were patients treated with oXiris^®^ at CRRT initiation. Controls were patients treated with standard AN69ST filters during the same period and under the same eligibility criteria. We performed 2:1 nearest-neighbor matching without replacement, matching each oXiris^®^-treated patient to up to 2 standard-filter controls by age, SOFA score at intensive care unit admission, time from intensive care unit admission to CRRT initiation, and infection source. Covariate balance before and after matching was assessed using standardized mean differences and is reported in **Table 1** and **Supplementary Figure 1**.

**Table 1:** Baseline demographic and clinical characteristics in the matched cohort, by treatment group.

Characteristic	Standard AN69ST (n=53)	oXiris^®^ (n=28)	Missing, n/N (%)	SMD
Age, years	62.0 [49.0–69.0]	65.5 [47.8–73.0]	0/81 (0.0)	0.03
Female sex, n (%)	16 (30.2)	10 (35.7)	0/81 (0.0)	0.12
Body mass index, kg/m²	28.1 [25.0–32.0]	26.5 [23.1–29.2]	0/81 (0.0)	0.53
Hypertension, n (%)	26 (49.1)	12 (42.9)	0/81 (0.0)	0.12
Diabetes mellitus, n (%)	19 (35.8)	7 (25.0)	0/81 (0.0)	0.24
Heart failure, n (%)	4 (7.5)	5 (17.9)	0/81 (0.0)	0.31
COPD, n (%)	3 (5.7)	4 (14.3)	0/81 (0.0)	0.29
SOFA score at ICU admission	8.0 [5.0–10.0]	8.0 [6.5–10.0]	0/81 (0.0)	0.00
COVID-19, n (%)	13 (24.5)	7 (25.0)	0/81 (0.0)	0.01
Sepsis source, n (%)			0/81 (0.0)	0.10
• Pulmonary	33 (62.3)	16 (57.1)		
• Abdominal	13 (24.5)	7 (25.0)		
• Urinary	5 (9.4)	3 (10.7)		
• Neurologic	0 (0.0)	0 (0.0)		
• Soft tissue	2 (3.8)	2 (7.1)		
Time to CRRT initiation, hours	3.0 [1.0–9.0]	2.0 [1.0–5.8]	0/81 (0.0)	0.03
PaO_2_/FiO_2_ at ICU admission, mm Hg	138.5 [80.5–227.2]	117.0 [70.5–192.2]	3/81 (3.7)	0.18
Lactate at ICU admission, mmol/L	2.3 [1.5–4.8]	4.7 [2.8–5.9]	19/81 (23.5)	0.23
Arterial pH at ICU admission	7.3 [7.2–7.4]	7.3 [7.3–7.4]	3/81 (3.7)	0.20
Mean arterial pressure at ICU admission, mm Hg	63.7 [57.0–79.3]	60.5 [52.5–74.2]	0/81 (0.0)	0.42

Values are median [interquartile range] or n (%). Missingness is reported as n/N (%). Standardized mean differences (SMDs) summarize post-matching balance and are shown for descriptive assessment rather than baseline hypothesis testing. Matching was performed 2:1 without replacement by nearest-neighbor propensity score matching on age, SOFA score at intensive care unit admission, time from intensive care unit admission to CRRT initiation, and infection source.

### 2.4 Interventions

All patients underwent continuous veno-venous hemodiafiltration with PrismaFlex devices (Baxter Healthcare, Deerfield, IL, USA). The prescribed effluent dose was 35 mL/kg/hour and could be adjusted according to hemodynamic tolerance and metabolic needs under local intensive care unit practice. Anticoagulation with unfractionated heparin was standard, whereas regional citrate anticoagulation was used when bleeding risk was judged higher. The oXiris^®^ filter (Baxter Healthcare, Lyon, France) is a surface-treated AN69-based membrane designed to provide renal support together with cytokine adsorption and enhanced endotoxin adsorption.

Supportive care, including antimicrobials, vasopressor support targeting a mean arterial pressure of at least 65 mm Hg, lung-protective ventilation when required, sedation, analgesia, and nutritional support, followed institutional protocols aligned with international sepsis guidelines. Because the retrospective database did not consistently capture delivered CRRT dose, number of filter exchanges, filter life, circuit clotting, crossover between membranes, fluid balance, antimicrobial timing, or source-control timing, these variables were not available for formal between-group comparison and are addressed as study limitations.

Filter selection was clinician-driven and influenced by bedside assessment of inflammatory burden, vasopressor requirement, metabolic severity, and device availability. We therefore treated membrane choice as a nonrandomized exposure with a high risk of confounding by indication and addressed it by design-stage matching, covariate-adjusted outcome modeling, and prespecified sensitivity analyses.

### 2.5 Data collection and variables

The primary outcome was 28-day all-cause mortality, defined as death from any cause within 28 days of intensive care unit admission. Intensive care unit admission was used as the primary analytic time zero because it was uniformly available for the full cohort and aligned with outcome ascertainment in the retrospective dataset. Exact timestamps necessary to perform a formal survival sensitivity analysis from CRRT initiation were not consistently available.

Secondary outcomes were ventilator-free days at day 28, intensive care unit–free days at day 28, CRRT duration, and intensive care unit length of stay. Ventilator-free days at day 28 were defined as 0 for patients who died before day 28; for survivors, ventilator-free days equaled 28 minus ventilated days, truncated at 28. Intensive care unit–free days at day 28 were defined similarly.

Baseline and serial physiologic variables available in the database included SOFA score, arterial lactate, PaO2/FiO2 ratio, arterial pH, mean arterial pressure, vasopressor dose, and C-reactive protein. To address reviewer concerns about early treatment-responsive endpoints in a high-mortality cohort, we prespecified exploratory paired-change analyses using the literal recorded data fields available in the matched cohort: SOFA 2d minus SOFA at admission, lactate 1d minus lactate at admission, PaO2/FiO2 3d minus PaO2/FiO2 at admission, mean arterial pressure 1d minus mean arterial pressure at admission, vasopressor dose 2d minus vasopressor dose 1d, and C-reactive protein 1d minus C-reactive protein at admission. These analyses were exploratory and intended to describe early physiologic trajectories rather than establish treatment efficacy.

C-reactive protein was measured in both treatment groups as part of routine sepsis care. Interleukin-6 and procalcitonin were obtained only in patients treated with oXiris^®^ under local testing practice and were not systematically measured in the standard-filter group. These biomarkers were therefore summarized descriptively within the oXiris^®^ cohort and were not used for formal between-group comparisons. Because the retrospective dataset did not uniformly capture mechanical ventilation status at CRRT initiation, source-control timing, antimicrobial timing, or fluid balance, these variables could not be included in formal comparative analyses.

### 2.6 Sample size

The target sample size was based on a reported mortality rate of 45.5% in sepsis-associated acute kidney injury and an assumed odds ratio of 3.88 for cytokine adsorption therapy (14). Under 80% power, a two-sided alpha of 0.05, and a 2:1 control-to-case ratio, 31 exposed patients and 62 controls were estimated to be required. The final eligible cohort met this numerical target. Because the observed mortality rate was higher than expected and the true treatment effect of an adjunctive extracorporeal therapy was likely smaller than the effect size assumed in the original calculation, the achieved sample should be interpreted as adequate for cohort assembly but limited for detecting moderate mortality differences.

### 2.7 Bias control

Selection bias was addressed by applying predefined eligibility criteria and identifying all consecutive eligible patients during the study period. Measurement bias was reduced through structured data abstraction and predefined variable coding. Confounding by indication was addressed through design-stage matching, prespecified covariate-adjusted outcome modeling, and weighted sensitivity analyses. Residual bias remained possible because treatment allocation was clinician-driven, several severity-sensitive baseline variables were incomplete or imbalanced after matching, and some co-interventions and CRRT operational details were not uniformly captured in the retrospective database.

### 2.8 Statistical analysis

All analyses were performed with R version 4.5.2 (15). Continuous variables are reported as median [interquartile range] and categorical variables as n (%). Missing codes 9999 and 8888 were recoded as missing before analysis. Missingness was quantified by variable and by treatment group, and there were no missing data for 28-day mortality. Analyses were based on complete cases for the variables included in each model. We did not perform multiple imputation because missingness was concentrated in selected baseline physiologic variables, the matched sample was modest, and *post hoc* imputation would have required additional assumptions that were not prespecified. Instead, we report variable-level missingness by group and interpret severity-sensitive analyses as exploratory.

To reduce confounding by indication, we constructed a 2:1 matched cohort using nearest-neighbor propensity score matching without replacement with the MatchIt package. The distance metric was logistic propensity score estimation, the matching ratio was 2 controls per treated patient, the caliper was 0.2 of the standard deviation of the logit of the propensity score, and no exact matching was used. Matching variables were age, SOFA score at intensive care unit admission, time from intensive care unit admission to CRRT initiation, and infection source category. Ties were handled by the software’s default nearest-neighbor routine for the package version used. Balance before and after matching was assessed with standardized mean differences and displayed in a Love plot.

The primary outcome was 28-day all-cause mortality. We estimated risk ratios and 95% confidence intervals with Poisson regression using a log link and sandwich standard errors. The prespecified minimal adjustment set included age, SOFA score at intensive care unit admission, and time from intensive care unit admission to CRRT initiation. Matching was used as design-stage preprocessing to improve baseline comparability rather than to create strictly paired inferential sets; therefore, treatment effects were estimated at the cohort level with prespecified covariate adjustment and robust standard errors. Kaplan-Meier curves are presented descriptively and should be interpreted as visual summaries rather than formal CRRT-anchored time-to-event analyses.

Secondary outcomes were analyzed as competing-risk–aware duration summaries. Ventilator-free days at day 28 and intensive care unit–free days at day 28 were reported both as continuous distributions and as binary indicators of more than 0 free days. For the binary secondary endpoints, adjusted risk ratios were estimated with the same Poisson model framework and prespecified covariates as in the primary analysis. Continuous CRRT duration and intensive care unit length of stay are reported descriptively.

We prespecified two sensitivity analyses. First, we repeated the primary model after excluding patients with COVID-19. Second, we performed inverse probability of treatment weighting with stabilized weights to target the average treatment effect. The propensity model for weighting included age, sex, hypertension, diabetes mellitus, heart failure, chronic obstructive pulmonary disease, human immunodeficiency virus infection, oncologic disease, autoimmune disease, COVID-19 status, infection source, and SOFA score at intensive care unit admission. Missingness in IPTW analyses was handled by complete-case restriction. Stabilized weights were truncated at the 1st and 99th percentiles, and post-weighting balance was assessed with standardized mean differences and a Love plot. Because the weighting analysis was applied to the matched analytic cohort, it was interpreted as a secondary sensitivity analysis rather than as a primary marginal effect estimate for the full eligible cohort.

Exploratory early physiologic endpoint analyses used paired within-patient changes between the literal recorded columns available in the matched cohort. For each endpoint, we report the number of paired observations, group-specific medians and interquartile ranges for change, and Wilcoxon rank-sum comparisons when the effective sample size was adequate. These analyses were exploratory, were not adjusted for multiplicity, and were not intended to establish efficacy.

Additional exploratory sensitivity analyses were performed in the matched cohort after further adjustment for admission lactate, mean arterial pressure, and PaO2/FiO2. A secondary exploratory model also included admission arterial pH. These models used complete cases for the included covariates and were intended to assess the consistency of the primary estimate under broader baseline severity adjustment. Multiple imputation was considered for selected baseline severity variables but was not retained because the sample size and missingness pattern did not support sufficiently reliable pooled inference.

### 2.9 Ethics

The study protocol was reviewed and approved by the CES Clinic Research Ethics Committee (Acta 053/Ae-749) and conducted in accordance with the principles of the Declaration of Helsinki (16). Written informed consent was obtained from all patients or their legally authorized representatives for the use of oXiris^®^ filters and data collection as part of routine clinical care. Clinical data were subsequently de-identified before retrospective analysis, and the study was classified as minimal risk in accordance with Colombian regulations (17).

## 3 Results

### 3.1 Participant flow

**Figure 1:**
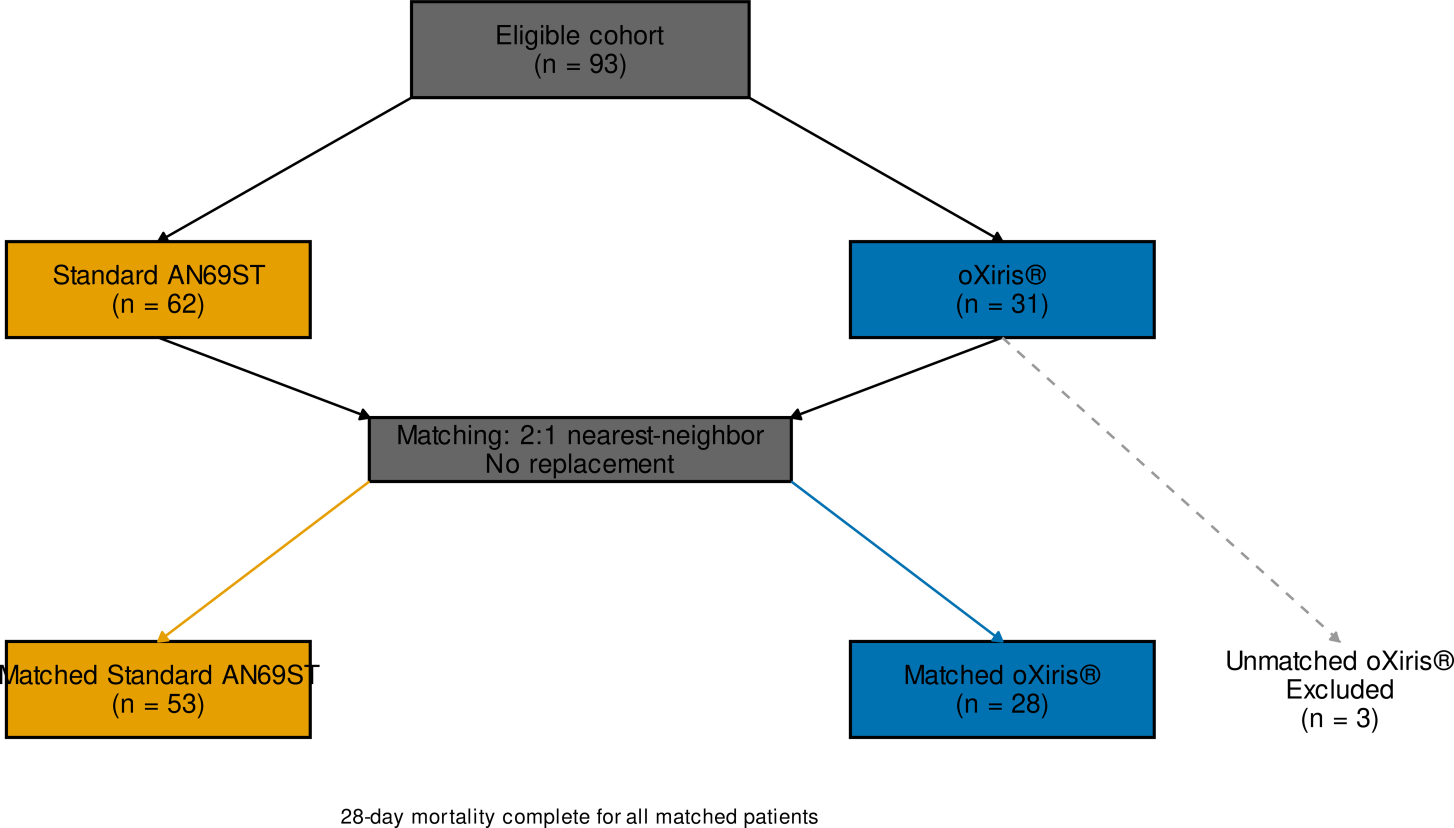
Cohort selection and matching flow. Among 93 consecutive eligible patients, 62 received standard AN69ST filters and 31 received oXiris^®^ at CRRT initiation. Nearest-neighbor 2:1 matching without replacement produced a matched cohort of 81 patients (53 standard AN69ST and 28 oXiris^®^). Three oXiris^®^-treated patients could not be matched. Twenty-eight-day mortality status was complete for all matched patients.

Between November 2020 and May 2023, 93 consecutive eligible patients met study criteria and entered the dynamic cohort: 62 received standard AN69ST filters and 31 received oXiris^®^ at CRRT initiation. After 2:1 nearest-neighbor matching without replacement, 81 patients were included in the matched cohort: 53 in the standard-filter group and 28 in the oXiris^®^ group. Three oXiris^®^-treated patients could not be matched because no suitable standard-filter controls were available under the prespecified matching strategy. There were no losses to follow-up for the primary outcome, and 28-day vital status was complete for all matched patients (**Figure 1**).

### 3.2 Baseline characteristics

Baseline characteristics of the matched cohort are summarized in **Table 1**. The prespecified matching variables—age, SOFA score at intensive care unit admission, time from intensive care unit admission to CRRT initiation, and infection source—showed close post-match balance. Several nonmatching variables remained clinically imbalanced, including body mass index and mean arterial pressure at admission, and baseline lactate and PaO2/FiO2 suggested greater physiologic severity in the oXiris^®^ group. Missingness was limited for most baseline variables but was more substantial for lactate and modest for PaO2/FiO2 and arterial pH. Because these residual differences and missing values could influence severity-sensitive analyses, they should be considered when interpreting both the primary comparison and the exploratory physiologic endpoints.

### 3.3 Primary outcome: 28-day mortality

**Figure 2:**
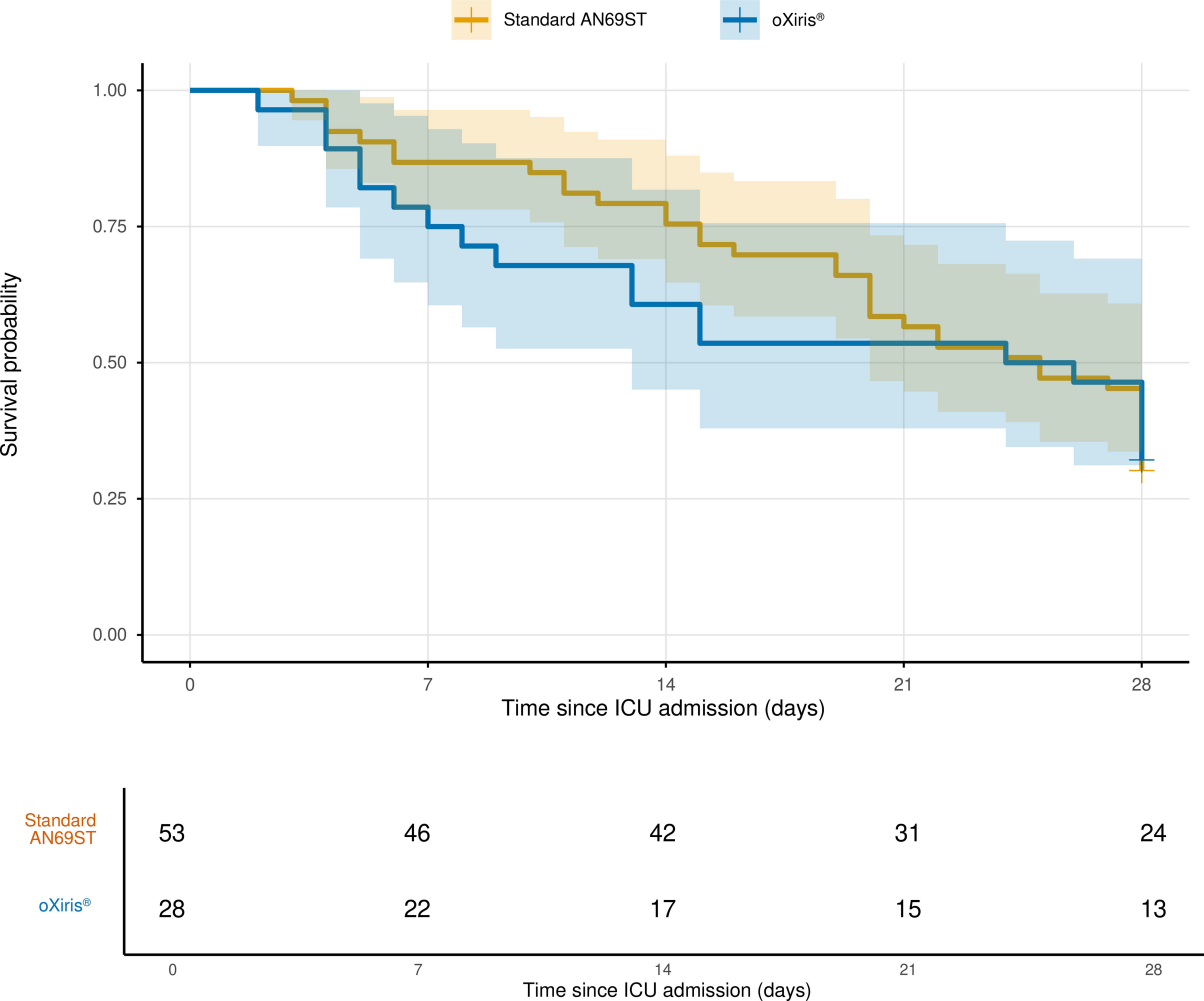
Descriptive 28-day survival in the matched cohort. Kaplan-Meier curves summarize 28-day survival from intensive care unit admission in the matched cohort. Because exact CRRT-initiation and event timestamps were not consistently available in the analytic dataset, this figure should be interpreted as a descriptive survival display rather than a formal CRRT-anchored time-to-event analysis.

In the matched cohort (n = 81), 28-day mortality was 69.8% (37/53) in the standard-filter group and 67.9% (19/28) in the oXiris^®^ group. In the adjusted Poisson model with sandwich standard errors, the risk ratio for oXiris^®^ versus standard filters was 0.95 (95% CI, 0.71–1.28; P = 0.76). The absolute risk difference was −2.0% (95% CI, −23.2% to 19.3%) (**Figure 2**).

### 3.4 Sensitivity analyses

After excluding patients with COVID-19 (n = 61), 28-day mortality was 62.5% (25/40) in the standard-filter group and 61.9% (13/21) in the oXiris^®^ group. The adjusted risk ratio was 1.05 (95% CI, 0.71-1.54; P = 0.81), which was directionally consistent with the primary analysis.

In the IPTW sensitivity analysis, stabilized average treatment effect weights were estimated within the matched analytic cohort using age, sex, hypertension, diabetes mellitus, heart failure, chronic obstructive pulmonary disease, human immunodeficiency virus infection, oncologic disease, autoimmune disease, COVID-19 status, infection source, and SOFA score at intensive care unit admission. Missingness in the weighting model was handled with complete-case restriction. After truncation of weights at the 1st and 99th percentiles, post-weighting balance was acceptable across all prespecified covariates by standardized mean-difference diagnostics. The weighted risk ratio for oXiris^®^ versus standard filters was 0.90 (95% CI, 0.63-1.31; P = 0.60), and the corresponding estimate after excluding COVID-19 cases was 0.93 (95% CI, 0.58-1.51; P = 0.78). Because weighting was applied to the matched cohort rather than the full eligible cohort, these analyses are presented as secondary sensitivity analyses.

In an additional exploratory complete-case analysis of the matched cohort, further adjustment for admission lactate, mean arterial pressure, and PaO2/FiO2 yielded a risk ratio for oXiris^®^ versus standard filters of 0.793 (95% CI, 0.568 to 1.108; P = 0.1749) in 62 patients (43 standard-filter and 19 oXiris^®^). A secondary exploratory model that also included admission arterial pH produced a similar estimate (risk ratio, 0.794; 95% CI, 0.568 to 1.110; P = 0.1767) and did not materially change interpretation. Multiple imputation was evaluated but was not performed because the available sample and missingness pattern did not support sufficiently reliable pooled inference.

Post-weighting balance diagnostics are shown in **Supplementary Table 2** and **Supplementary Figure 2**.

### 3.5 Secondary outcomes

Secondary outcomes are summarized in **Table 2**. Ventilator-free days at day 28 and intensive care unit–free days at day 28 were calculated with competing-risk–aware definitions that assign 0 free days to patients who died before day 28. Median ventilator-free days were 0.0 [0.0-0.0] in the standard-filter group and 0.0 [0.0-3.8] in the oXiris^®^ group; 11 of 53 patients (20.8%) and 7 of 28 patients (25.0%), respectively, had more than 0 ventilator-free days. Median intensive care unit–free days were 0.0 [0.0-0.0] with standard filters and 0.0 [0.0-1.0] with oXiris^®^; 11 of 53 patients (20.8%) and 7 of 28 patients (25.0%), respectively, had more than 0 intensive care unit–free days. In adjusted Poisson models for the binary more-than-0 free-day outcomes, the risk ratio for oXiris^®^ versus standard filters was 0.82 (95% CI, 0.37-1.81) for ventilator-free days and 0.82 (95% CI, 0.37-1.81) for intensive care unit–free days. Given the concentration of values at 0 in both groups, these endpoints should be interpreted as exploratory summaries of resource use in a cohort with very high early mortality.

**Table 2:** Clinical outcomes in the matched cohort by hemofilter group.

Outcome	Standard AN69ST (n=53)	oXiris^®^ (n=28)	Effect estimate (95% CI)	Additional information
28-day mortality, n/N (%)	37/53 (69.8)	19/28 (67.9)	Adjusted RR 0.95 (0.71–1.28)	RD −2.0% (−23.2% to 19.3%)
Ventilator-free days at day 28, median [IQR]	0.0 [0.0–0.0]	0.0 [0.0–3.8]	Descriptive	—
Ventilator-free days at day 28 >0, n/N (%)	11/53 (20.8)	7/28 (25.0)	Adjusted RR 0.82 (0.37–1.81)	—
ICU-free days at day 28, median [IQR]	0.0 [0.0–0.0]	0.0 [0.0–1.0]	Descriptive	—
ICU-free days at day 28 >0, n/N (%)	11/53 (20.8)	7/28 (25.0)	Adjusted RR 0.82 (0.37–1.81)	—
CRRT duration, days, median [IQR]	6.0 [4.0–11.0]	4.0 [2.8–7.0]	Descriptive	—
ICU length of stay, days, median [IQR]	20.0 [11.0–27.0]	13.0 [6.8–24.5]	Descriptive	—

Adjusted risk ratios were estimated with Poisson regression using a log link and sandwich standard errors, adjusted for age, SOFA score at intensive care unit admission, and time from intensive care unit admission to CRRT initiation. Ventilator-free days and intensive care unit–free days at day 28 assign 0 free days to patients who died before day 28; among survivors, free days equal 28 minus ventilated days or intensive care unit length of stay, truncated at 28. Continuous duration variables are descriptive. Binary more-than-0 free-day analyses are exploratory.

Missingness by variable and by treatment group is summarized in **Table 1** and **Supplementary Table 3**. Admission lactate had the greatest baseline missingness among key severity markers (22/93, 23.66%), whereas 28-day mortality was complete for all matched patients.

### 3.6 Exploratory early physiologic trajectories

**Figure 3:**
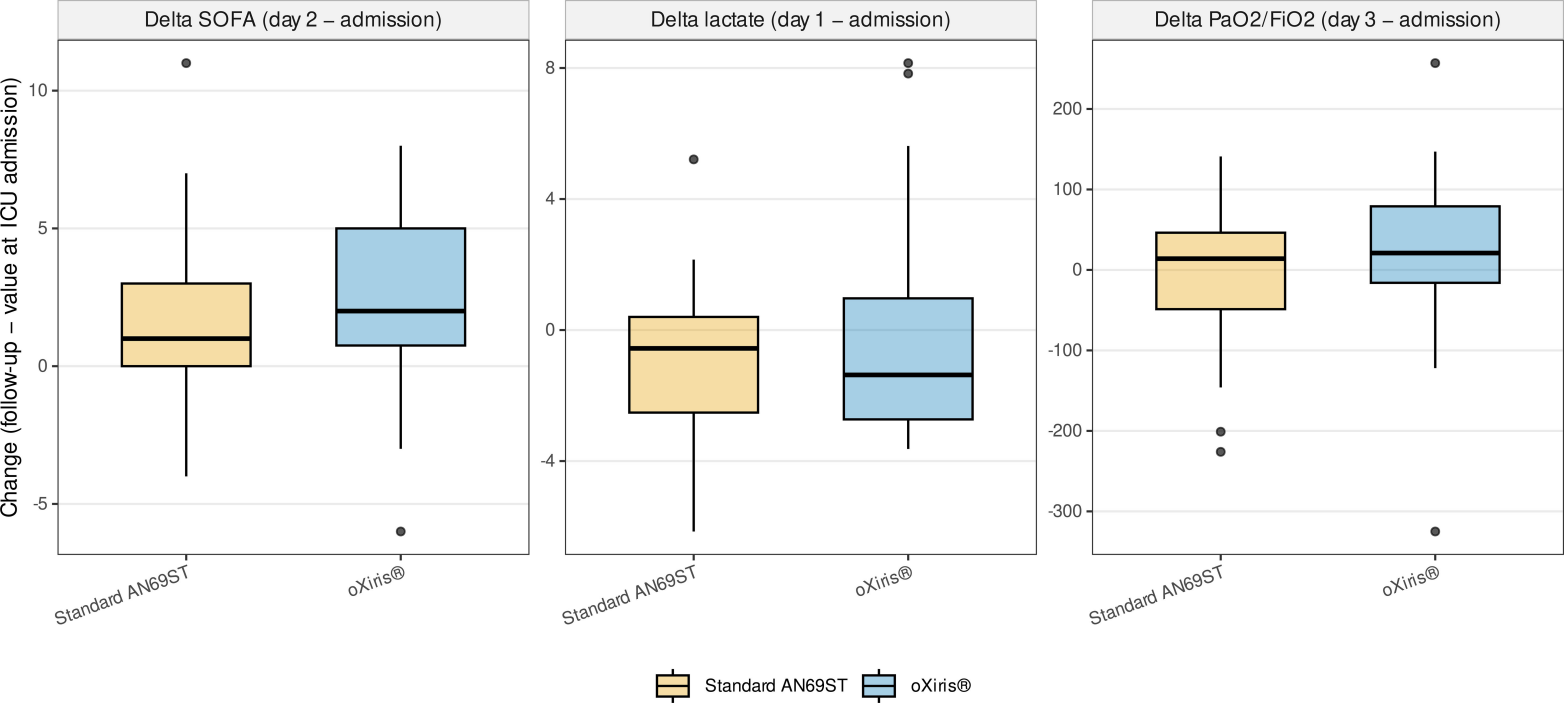
Exploratory early physiologic summaries in the matched cohort. Panels summarize early recorded SOFA, lactate, and PaO2/FiO2 values in the matched cohort. Because the retrospective database used variable-specific recorded time points, these curves are descriptive summaries and should be interpreted together with **Table 3**, which reports paired available-case changes using the literal database columns.

To address early treatment-responsive physiologic endpoints in this high-mortality cohort, we examined paired changes using the literal recorded time points available in the matched dataset. Median change in SOFA score from admission to SOFA 2d was +1 [0 to 3] in the standard-filter group and +2 [0.75 to 5] in the oXiris^®^ group (P = 0.12). Median change in lactate from admission to lactate 1d was -0.56 mmol/L [-2.52 to 0.40] in the standard-filter group and -1.37 mmol/L [-2.73 to 0.97] in the oXiris^®^ group (P = 0.81). Median change in PaO2/FiO2 from admission to PaO2/FiO2 3d was +14 mm Hg [-48.75 to 46.25] in the standard-filter group and +21 mm Hg [-16 to 79] in the oXiris^®^ group (P = 0.38) (**Figure 3**).

**Table 3:** Exploratory paired early physiologic changes in the matched cohort, by hemofilter group.

Endpoint definition	Delta computation	Standard AN69ST, n	Standard AN69ST median [IQR]	oXiris^®^, n	oXiris^®^ median [IQR]	P value
SOFA admission vs SOFA 2d	SOFA 2d - SOFA admission	53	1 [0 to 3]	28	2 [0.75 to 5]	0.12
Lactate admission vs lactate 1d, mmol/L	Lactate 1d - lactate admission	27	-0.56 [-2.52 to 0.40]	16	-1.37 [-2.73 to 0.97]	0.81
PaO2/FiO2 at ICU admission vs PaO2/FiO2 3d, mm Hg	PaO2/FiO2 3d – PaO2/FiO2 at ICU admission	52	14 [-48.75 to 46.25]	25	21 [-16 to 79]	0.38
Mean arterial pressure admission vs MAP 1d, mm Hg	MAP 1d - MAP admission	53	-2.34 [-11 to 3]	28	-1.50 [-12.08 to 8.75]	0.75
Maximum vasopressor dose 1d vs 2d	Dose 2d - Dose 1d	52	0.00 [-0.09 to 0.03]	26	0.00 [-0.10 to 0.10]	0.44
C-reactive protein admission vs CRP 1d, mg/dL	CRP 1d - CRP admission	31	1.41 [-3.95 to 4.30]	17	0.20 [-1.50 to 4.57]	0.93

Deltas were calculated from literal recorded database columns within paired available cases only. P values are from Wilcoxon rank-sum comparisons of change distributions between groups and are exploratory. These analyses were not adjusted for multiplicity and should be interpreted in the context of missingness and residual baseline imbalance.

Additional exploratory paired changes did not show clear between-group differences. Median change in mean arterial pressure from admission to mean arterial pressure 1d was -2.34 mm Hg [-11 to 3] in the standard-filter group and -1.5 mm Hg [-12.08 to 8.75] in the oXiris^®^ group (P = 0.75). Median change in maximum vasopressor dose from dose 1d to dose 2d was 0.00 [-0.09 to 0.03] in the standard-filter group and 0.00 [-0.10 to 0.10] in the oXiris^®^ group (P = 0.44). Median change in C-reactive protein from admission to CRP 1d was +1.41 mg/dL [-3.95 to 4.30] in the standard-filter group and +0.20 mg/dL [-1.50 to 4.57] in the oXiris^®^ group (P = 0.93) (**Table 3**). These analyses were exploratory, based on paired available cases only, and should be interpreted in the context of missingness and residual baseline imbalance.

## 4 Discussion

In this retrospective matched cohort of adults with septic shock and sepsis-associated acute kidney injury requiring continuous renal replacement therapy, oXiris^®^ use was not associated with lower 28-day mortality than standard AN69ST filters. The primary matched analysis, the COVID-19 exclusion sensitivity analysis, and the IPTW sensitivity analysis were directionally consistent. Because the weighting analysis was performed within the matched analytic cohort rather than the full eligible cohort, we interpret IPTW as a secondary sensitivity analysis rather than as a coequal primary estimate.

Our results need to be interpreted in the context of emerging syntheses of the oXiris^®^ literature. A recent meta-analysis that pooled 14 observational studies and randomized trials reported lower 28-day mortality and shorter ICU stays in sepsis patients treated with oXiris^®^ compared with other CRRT filters, along with reductions in SOFA score, norepinephrine dose, IL-6, and lactate levels (12). At the same time, the authors rated the certainty of evidence as low or very low across outcomes due to the predominance of nonrandomized designs and unclear risk of bias in the randomized trials, a concern that was reinforced in a subsequent commentary (18). A second systematic review focused on IL-6 kinetics also found consistent reductions in IL-6 concentrations and improvements in vasoactive-inotropic score, SOFA, procalcitonin, and CRP with oXiris^®^ but did not establish a clear survival effect (19). In a broader network meta-analysis of adsorptive blood purification modalities, oXiris^®^ ranked favorably for lactate reduction but not among the top devices for mortality or ICU length of stay when compared with polymyxin B hemoperfusion, HA330 cartridges, and other techniques (20). The absence of a mortality signal in our cohort is therefore compatible with an evidence base that supports biological activity and short-term physiological effects of oXiris^®^ but remains inconclusive regarding survival benefit.

Duration-based outcomes in septic shock are strongly influenced by early death. We addressed this by using ventilator-free days and intensive care unit–free days at day 28, which assign 0 free days to patients who die before day 28. In our matched cohort, both outcomes were concentrated at 0, which limited their ability to discriminate between treatment groups and supports interpreting them as exploratory rather than decisive efficacy endpoints.

We also added exploratory early physiologic analyses to address reviewer concerns that conventional duration-based outcomes can be uninformative in cohorts with mortality near 70%. Paired changes in recorded SOFA, lactate, PaO2/FiO2, mean arterial pressure, vasopressor dose, and C-reactive protein did not show clear between-group differences. These analyses are useful as descriptive adjuncts, but they do not overcome the core limitations of a nonrandomized study with residual imbalance and incomplete physiologic data.

Strengths of this study include consecutive patient identification within a defined intensive care unit cohort, a clearly specified matched design, complete ascertainment of the primary outcome in the matched cohort, and reproducible primary and sensitivity analyses. The matching strategy improved balance in the prespecified design variables, and weighted sensitivity analyses showed acceptable post-weighting balance across measured covariates within the matched analytic cohort.

This study also has important limitations. First, the single-center design and modest sample size limit precision, and the confidence intervals do not exclude clinically relevant benefit or harm. The study was powered under assumptions that proved optimistic for an adjunctive extracorporeal intervention in severe septic shock, which left limited power to detect moderate mortality differences. Second, treatment allocation was clinician-driven and influenced by perceived inflammatory or hemodynamic severity together with device availability. Residual confounding remains plausible because several clinically relevant baseline variables, including body mass index, mean arterial pressure, lactate, and PaO2/FiO2, were not used in matching and remained imbalanced or incomplete after matching. Third, missingness was nontrivial for selected physiologic variables, particularly lactate and serial biomarkers. We addressed this by reporting missingness explicitly by group and restricting analyses to complete cases, but complete-case analyses can still introduce selection and reduce precision.

Additional exploratory models incorporating baseline lactate, mean arterial pressure, and PaO2/FiO2 yielded estimates that were consistent with the primary analysis and did not suggest a materially different treatment effect. These analyses provide some reassurance that the primary result was not driven solely by the original minimal adjustment set, but they remain limited by reduced effective sample size, residual confounding, and selection related to incomplete baseline physiologic data. A secondary model that also included arterial pH was directionally similar. Multiple imputation was considered but was not adopted because the available sample and missingness structure did not support sufficiently reliable pooled estimates.

Fourth, exact timestamps required for a formal CRRT-initiation survival sensitivity analysis were not consistently available, so intensive care unit admission was used as the primary analytic time zero. Fifth, the retrospective database did not systematically capture crossover between filter types, number of sequential filter exchanges, filter life, circuit clotting, delivered CRRT dose, fluid balance, antimicrobial timing, source-control timing, or baseline mechanical ventilation status for formal group comparison. Sixth, interleukin-6 and procalcitonin were not routinely measured in the standard-filter group, which precluded formal between-group biomarker comparisons and limited mechanistic inference. Seventh, the IPTW analysis was performed on the matched cohort rather than on the full eligible cohort, so it should be viewed as a sensitivity analysis, not as a primary marginal treatment-effect estimate. Finally, the exploratory early physiologic endpoints were based on literal recorded time points in the database, were not adjusted for multiplicity, and should not be interpreted as proof of differential biologic response.

Because filter choice was clinician-driven and influenced by perceived severity and availability, confounding by indication remains a central threat to inference. If oXiris^®^ was selected for patients thought to be at higher risk, bias would favor the standard-filter group; if it was selected for patients thought more likely to recover, bias would favor oXiris^®^. Matching, covariate adjustment, and weighted sensitivity analyses reduced measured imbalance but cannot remove unmeasured confounding or time-varying treatment decisions.

In summary, in this matched cohort of adults with septic shock and sepsis-associated acute kidney injury requiring continuous renal replacement therapy, oXiris^®^ use was not associated with lower 28-day mortality than standard AN69ST filters. Ventilator-free days and intensive care unit–free days were heavily constrained by early mortality, and exploratory early physiologic changes did not show a clear between-group signal. These findings should be interpreted in the setting of clinician-driven treatment selection, residual baseline imbalance, incomplete physiologic and biomarker data, and limited CRRT operational detail. Larger randomized studies with standardized co-interventions, clearly anchored time-zero definitions, and prespecified mechanistic and clinical endpoints are needed to determine whether any subgroup benefits from oXiris^®^-based therapy.

## 5 Conclusions

In this retrospective matched cohort of adults with septic shock and sepsis-associated acute kidney injury requiring continuous renal replacement therapy, oXiris^®^ use was not associated with lower 28-day mortality than standard AN69ST filters. Competing-risk–aware secondary outcomes and exploratory early physiologic analyses did not show a clear between-group advantage and should not be overinterpreted. These data do not establish a survival benefit for oXiris^®^ in this setting. Future randomized studies should incorporate standardized co-interventions, explicit CRRT-anchored timing, and prespecified clinical and mechanistic endpoints.

## Data Availability

The raw data supporting the conclusions of this article will be made available by the authors, without undue reservation.
