# Supplementary material for: Use of oXiris® vs standard AN69ST filters in sepsis-associated acute kidney injury requiring continuous renal replacement therapy: a retrospective matched cohort study
Source: Front Nephrol. 2026 May 12;6:1810007. doi: 10.3389/fneph.2026.1810007 (PMC13201489; doi:10.3389/fneph.2026.1810007)
Supplement: Supplementary file 1 [file SupplementaryFile1.docx]

Supplementary Material

# Supplementary Tables

**Supplementary Table S1. Crude Outcome Estimates.**

| Outcome | Standard AN69ST (n = 53) | oXiris^®^ (n = 28) | Unadjusted Effect Estimate (95% CI) | Notes |
| --- | --- | --- | --- | --- |
| 28-day mortality | 37/53 (69.8%) | 19/28 (67.9%) | RR 0.97 (0.71–1.33) | RD -2.0% (-23.2% to 19.3%) |
| Ventilator-free days at day 28 | 0.0 [0.0–0.0] | 0.0 [0.0–3.8] | 0.0 (0.0–0.0) | Hodges-Lehmann |
| Patients with >0 ventilator-free days at day 28 | 11/53 (20.8%) | 7/28 (25.0%) | RR 1.20 (0.53–2.76) | — |
| ICU-free days at day 28 | 0.0 [0.0–0.0] | 0.0 [0.0–1.0] | 0.0 (0.0–0.0) | Hodges-Lehmann |
| Patients with >0 ICU-free days at day 28 | 11/53 (20.8%) | 7/28 (25.0%) | RR 1.20 (0.53–2.76) | — |
| CRRT duration, days | 6.0 [4.0–11.0] | 4.0 [2.8–7.0] | -2.0 (-4.0 to -1.0) | Hodges-Lehmann |
| ICU length of stay, days | 20.0 [11.0–27.0] | 13.0 [6.8–24.5] | -5.0 (-10.0 to 1.0) | Hodges-Lehmann |

*Values are median [interquartile range] or n/N (%). Unadjusted risk ratios are shown for binary outcomes. For continuous outcomes, the unadjusted effect estimate is the Hodges-Lehmann location shift. Ventilator-free days and ICU-free days at day 28 assign 0 free days to patients who died before day 28. RR indicates risk ratio; RD, risk difference; ICU, intensive care unit; CRRT, continuous renal replacement therapy.*

**Supplementary Table S2. Standardized Mean Differences Before and After IPTW in the Matched Analytic Cohort.**

| Variable | Unweighted SMD | Weighted SMD | Balanced After Weighting |
| --- | --- | --- | --- |
| Age | -0.029 | -0.019 | Yes |
| Male sex | -0.055 | -0.019 | Yes |
| Hypertension | -0.062 | -0.042 | Yes |
| Diabetes mellitus | -0.108 | -0.010 | Yes |
| Heart failure | 0.103 | 0.008 | Yes |
| Chronic obstructive pulmonary disease | 0.086 | -0.001 | Yes |
| Human immunodeficiency virus infection | 0.086 | 0.002 | Yes |
| Oncologic disease | -0.044 | -0.023 | Yes |
| Autoimmune disease | -0.006 | 0.004 | Yes |
| COVID-19 | 0.005 | -0.018 | Yes |
| Sepsis source: pulmonary | -0.051 | -0.058 | Yes |
| Sepsis source: abdominal | 0.005 | 0.056 | Yes |
| Sepsis source: urinary | 0.013 | 0.000 | Yes |
| Sepsis source: soft tissue | 0.034 | 0.002 | Yes |
| SOFA score at ICU admission | 0.002 | 0.017 | Yes |

*IPTW indicates inverse probability of treatment weighting; SMD, standardized mean difference; SOFA, Sequential Organ Failure Assessment. Weighted estimates were obtained with stabilized average treatment effect weights in the matched analytic cohort. Covariates with an absolute weighted SMD below 0.10 were considered adequately balanced.*

**Supplementary Table S3. Missingness by Variable and Treatment Group.**

| Variable | Total Missing, n/N (%) | Standard AN69ST Missing, n/N (%) | oXiris^®^ Missing, n/N (%) |
| --- | --- | --- | --- |
| Age | 0/93 (0.0%) | 0/62 (0.0%) | 0/31 (0.0%) |
| Sex | 0/93 (0.0%) | 0/62 (0.0%) | 0/31 (0.0%) |
| Body mass index | 0/93 (0.0%) | 0/62 (0.0%) | 0/31 (0.0%) |
| Hypertension | 0/93 (0.0%) | 0/62 (0.0%) | 0/31 (0.0%) |
| Diabetes mellitus | 0/93 (0.0%) | 0/62 (0.0%) | 0/31 (0.0%) |
| Heart failure | 0/93 (0.0%) | 0/62 (0.0%) | 0/31 (0.0%) |
| Chronic obstructive pulmonary disease | 0/93 (0.0%) | 0/62 (0.0%) | 0/31 (0.0%) |
| Human immunodeficiency virus infection | 0/93 (0.0%) | 0/62 (0.0%) | 0/31 (0.0%) |
| Oncologic disease | 0/93 (0.0%) | 0/62 (0.0%) | 0/31 (0.0%) |
| Autoimmune disease | 0/93 (0.0%) | 0/62 (0.0%) | 0/31 (0.0%) |
| SOFA at ICU admission | 0/93 (0.0%) | 0/62 (0.0%) | 0/31 (0.0%) |
| COVID-19 | 0/93 (0.0%) | 0/62 (0.0%) | 0/31 (0.0%) |
| Sepsis source | 0/93 (0.0%) | 0/62 (0.0%) | 0/31 (0.0%) |
| Time from ICU admission to CRRT initiation | 0/93 (0.0%) | 0/62 (0.0%) | 0/31 (0.0%) |
| PaO2/FiO2 at ICU admission | 3/93 (3.23%) | 1/62 (1.61%) | 2/31 (6.45%) |
| Lactate at ICU admission | 22/93 (23.66%) | 13/62 (20.97%) | 9/31 (29.03%) |
| Arterial pH at ICU admission | 3/93 (3.23%) | 1/62 (1.61%) | 2/31 (6.45%) |
| Mean arterial pressure at ICU admission | 0/93 (0.0%) | 0/62 (0.0%) | 0/31 (0.0%) |
| Anticoagulation | 0/93 (0.0%) | 0/62 (0.0%) | 0/31 (0.0%) |
| CRRT duration | 0/93 (0.0%) | 0/62 (0.0%) | 0/31 (0.0%) |

*Percentages are based on the full eligible cohort (n = 93), including 62 patients treated with standard AN69ST filters and 31 treated with oXiris®. ICU indicates intensive care unit; CRRT, continuous renal replacement therapy; SOFA, Sequential Organ Failure Assessment.*

# Supplementary Figures

**Supplementary Figure S1. Standardized mean differences before and after propensity score matching.**

**
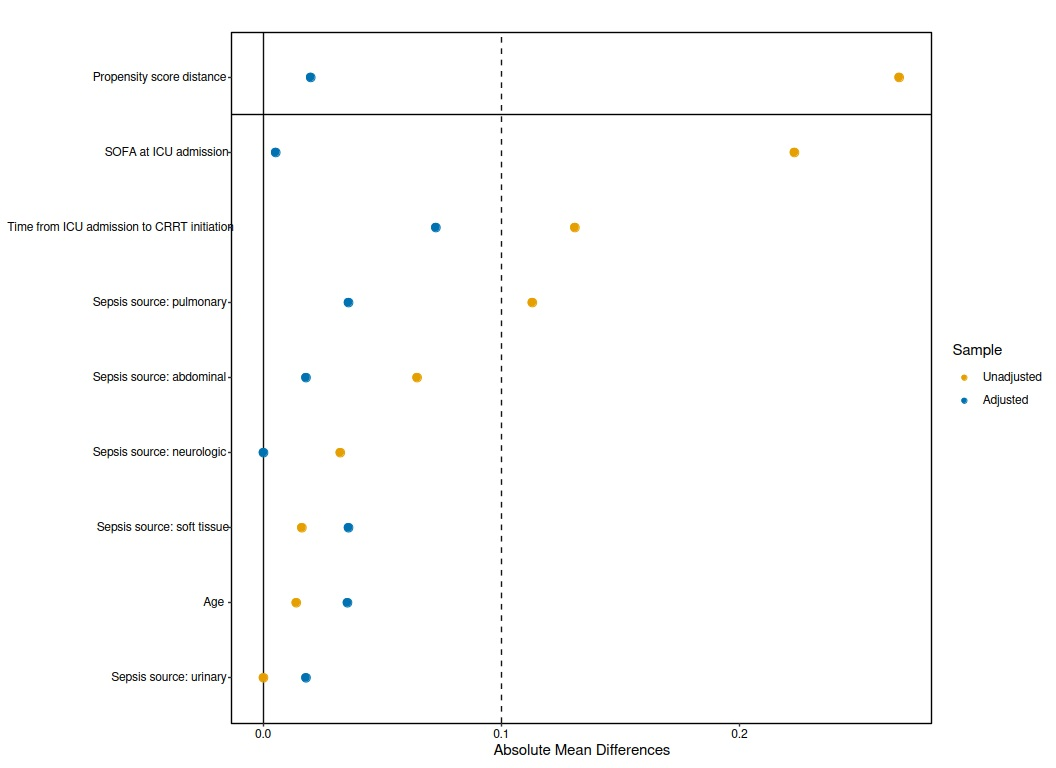
**

*Love plot showing absolute standardized mean differences for the variables used in the matching procedure before and after 2:1 nearest-neighbor propensity score matching without replacement. Matching was performed on age, SOFA score at ICU admission, time from ICU admission to CRRT initiation, and sepsis source. The vertical dashed line marks the prespecified balance threshold of 0.10. Lower values indicate better covariate balance after matching. SOFA indicates Sequential Organ Failure Assessment; ICU, intensive care unit; CRRT, continuous renal replacement therapy.*

**Supplementary Figure S2. Standardized mean differences before and after inverse probability of treatment weighting in the matched analytic cohort.**

**
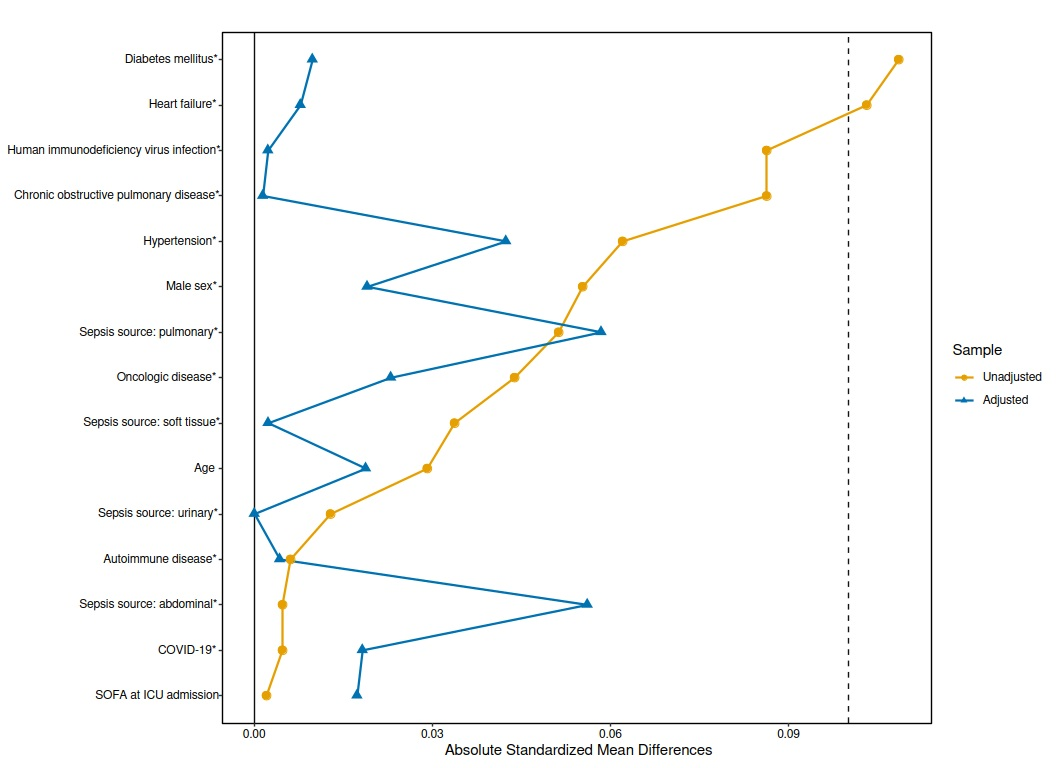
**

*Love plot showing absolute standardized mean differences before and after inverse probability of treatment weighting in the matched analytic cohort. Stabilized average treatment effect weights were estimated using baseline demographics, comorbidities, COVID-19 status, sepsis source, and SOFA score at ICU admission. The vertical dashed line marks the prespecified balance threshold of 0.10. Lower values indicate better covariate balance after weighting. Because weighting was applied to the matched analytic cohort, this figure should be interpreted as a secondary sensitivity analysis rather than as a full-cohort marginal treatment-effect analysis. SOFA indicates Sequential Organ Failure Assessment; ICU, intensive care unit.*
